# Evaluation of Auramine O staining and conventional PCR for leprosy diagnosis: A comparative cross-sectional study from Ethiopia

**DOI:** 10.1371/journal.pntd.0006706

**Published:** 2018-09-04

**Authors:** Selfu Girma, Charlotte Avanzi, Kidist Bobosha, Kassu Desta, Munir H. Idriss, Philippe Busso, Yohannes Tsegaye, Shimelis Nigusse, Tsegaye Hailu, Stewart T. Cole, Abraham Aseffa

**Affiliations:** 1 Armauer Hansen Research Institute, Addis Ababa, Ethiopia; 2 Global Health Institute, École Polytechnique Fédérale de Lausanne, Lausanne, Switzerland; 3 Addis Ababa University, CHS, Department of Medical Laboratory Sciences, Addis Ababa, Ethiopia; 4 ALERT hospital, Addis Ababa, Ethiopia; 5 Institut Pasteur, Paris, France; King Saud University College of Medicine, SAUDI ARABIA

## Abstract

**Background:**

Diagnosis of leprosy mainly relies on clinical examination due to the inconsistent sensitivity and poor reproducibility of the current laboratory tests. Utilisation of alternative methods to the standard Ziehl Neelsen (ZN), Fite-Faraco (FF) and Haematoxylin and Eosin (H&E) staining procedures may eventually improve leprosy diagnosis.

**Methodology/Principal findings:**

In this comparative study, the performance of the fluorescent Auramine O (AO) staining and polymerase chain reaction (PCR) was assessed with different skin samples using a combination of ZN, FF and H&E staining as the gold standard. AO, ZN, FF, H&E and PCR tests were performed on slit skin smears (SSS) and/or punch biopsies collected from 141 clinically confirmed leprosy cases and 28 non-leprosy skin samples. DNA was extracted from punch biopsies using two different methods with or without mechanical lysis.

Sensitivities were 87.6%, 59.3% and 77% for H&E, ZN and FF, respectively, whereas it reached 65.5% and 77.9% for AO in SSS and tissue sections and 91.1% for PCR in tissue samples. Morover, samples with low bacillary index, sensitivity of AO staining (61.8%) was similar to FF (60%, *p>*0.05) and lower than PCR (86.6%, *p*<0.05). Sensitivity of PCR also increased (96.8%, *p*<0.05) when mechanical lysis was used during DNA extraction compared to enzymatic treatment alone (84.6%).

**Conclusions/Significance:**

Our results showed that for diagnostic purposes, analysis of skin section is more sensitive than SSS, especially for samples with low bacillary load. AO staining on SSS and tissue sections was not significantly better than other routine diagnostic tests but considerably more user friendly. The sensitivity of PCR was higher than current standard methods and increased when combined with more efficient DNA extraction using mechanical and chemical lysis. Therefore, we recommend AO staining for the diagnosis of leprosy in lower health facilities such as health centres and district hospitals and PCR diagnosis at referral level and research centres.

## Author summary

Leprosy is one of the oldest reported infectious diseases in the history of humanity, with a remarkably high rate of active transmission still reported annually, but mainly in resource-limited endemic countries. The primary tool for leprosy transmission prevention is diagnosing the disease at an early stage. However, this is challenging in resource-limited disease-endemic countries with the currently available microscopic diagnostic tests. We found that Auramine O staining can alternatively be used for leprosy diagnosis using light-emitting diode fluorescence microscopy on both in slit skin smears and tissue section samples with a potential of replacing the routine light microscopic examination. We have also showed that a combination of highly efficient DNA extraction steps with a single run of conventional PCR out performed the standard microscopy methods for all samples with high and low bacillary index.

## Introduction

*Mycobacterium leprae* is the causative agent of leprosy, a chronic granulomatous infectious disease affecting the skin and peripheral nerves [1]. Leprosy manifests in various forms based on the immunological profiles and bacterial load in patients [1]. According to Ridley and Jopling, leprosy is classified as indeterminate (IND), tuberculoid (TT), borderline tuberculoid (BT), borderline (BB), borderline lepromatous (BL) and lepromatous leprosy (LL) [2]. More recently, for therapy purposes, the World Health Organization (WHO) implemented another classification depending on the number of lesions. Patients with 5 or less skin lesions are considered as paucibacillary (PB) cases and are treated for six months with two antibiotics whereas those with 6 or more lesions are regarded as multibacillary (MB) and receive three drugs for one year [3,4].

In 2016, about 214,783 new cases of leprosy were reported worldwide [5] including 19,384 (9%) in Africa. With 3,692 new cases, Ethiopia was the second highest African countries with respect to leprosy prevalence. The trend of new cases reported for the last ten years is stable with 4,086 per year on average. This data indicated the ongoing active transmission despite intense efforts to eliminate leprosy as a public health problem and the widespread use of multidrug therapy (MDT) [6]. Lack of reliable diagnostic tools especially for the early stage of the disease is of major concern [7,8]. Hence efforts to improve diagnosis are being undertaken and WHO has also set early detection of leprosy as a priority in leprosy control strategy [9,10].

Since *M*. *leprae* cannot be cultivated *in vitro*, clinical signs such as presence of lesions, sensory loss, and thickened peripheral nerves serve as the primary tool of leprosy diagnosis. However, the disease can easily be confused with other skin pathologies indicating the need for a differential leprosy diagnosis especially by less experienced physicians [3,5,11]. For this reason, along with clinical examination, identification of the bacteria and the histopathological classification in skin samples is necessary to confirm leprosy diagnosis. The most popular tools are Ziehl-Neelsen (ZN) and Fite-Faraco (FF) staining performed on clinical samples such as slit-skin smears (SSS), nasal swabs and formalin fixed paraffin embedded (FFPE) tissue samples [12–16]. Even though ZN and FF are available at lower level health institutions of resource-limited countries, their performance indetecting *M*. *leprae* bacilli is low, particularly in PB patients [17]. Therefore, for these problematic cases, clinician mostly relies on clinical examination which requires experience. In return, this highlights the need for more sensitive techniques to support clinical diagnosis. Auramine O (AO) staining is a fluorescence-based method widely used to detect mycobacterial species such as *M*. *tuberculosis* and *M*. *leprae* [18,19]. AO has been previously evaluated to be more sensitive for *M*. *leprae* detection in tissue sections compared to FF and is less time-consuming [20,21]. Molecular methods such as conventional PCR are even more sensitive, and can help with leprosy diagnosis [13,22–25]. However, such techniques are not widely available. Hence, this study was designed to determine the diagnostic utility of AO staining and conventional PCR in routine diagnosis in comparison with the standard protocol.

## Methods

### Ethical consideration

Ethical clearance was obtained from the Armauer Hansen Research Institute/All African Leprosy, Tuberculosis and Rehabilitation AHRI/ALERT Ethical Review Committee, Addis Ababa University College of Health Sciences, Department of Medical Laboratory Sciences Ethics and Research Review Committee and National Research Ethics Review Committee. Written informed consent was obtained from participants and parents or guardians of participating children.

### Study population

A total of 141 leprosy cases comprising 136 newly diagnosed treatment naïve and five relapse leprosy patients with any form of the disease were enrolled in this prospective comparative cross-sectional study at the ALERT center from January 2015 to April 2016. All cases were clinically diagnosed and confirmed by a dermatologist. Non-leprosy patients (n = 28) visiting the minor surgery department of the ALERT hospital were enrolled in the study as a control group. These patients did not present signs of leprosy.

### Data and sample collection

#### Sociodemographic and clinical parameters

Nurses collected sociodemographic data and clinical information of study participants using a structured questionnaire at the ALERT Red Medical Clinic before the participants went to the sample collection area. Participants in the control group were recruited before their admission for routine surgical treatment.

#### Sample collection

Slit skin smears (SSS) were collected from three different body sites (right and left earlobes, and either an eyebrow, the forehead or one of the arms) of leprosy patients to increase the probability of detecting acid-fast bacilli. While collecting SSS for routine ZN diagnosis, a duplicate slide was prepared from the same site at the same time and sent to the AHRI pathology laboratory for AO staining [26].

One 6mm skin punch sample was collected by well-trained nurses for each leprosy patient. Punch biopsy collection was not performed on cosmetic and sensitive body parts like the face and scrotal area [27,28]. For the non-leprosy control group, skin biopsy samples of ~10 mm diameter were collected from discarded skin specimens after routine surgical treatment. After collection, each punch biopsy was divided into two parts; one was placed in 10% buffered formalin solution to be processed for staining and the other in 70% ethanol for DNA extraction.

### Sample processing

#### ZN and AO staining on SSS

One of the slides containing SSS was dried for 15 min at room temperature and fixed from below by passing slowly through the flame of a spirit burner three times. The slide was then stained with 1% carbolfuchsin solution, heated as above until vapor begins to rise for 5 min. The slide was then washed with running tap water and destained with 1% acid-alcohol for 10–20 s, rinsed with tap water gently, counterstained with 0.2% methylene blue for 1min, washed again with tap water and air dried. Finally, it was examined under a 100X objective of a conventional light microscope [26].

For AO staining, the slide was flooded with 0.1% AO (MERCK, Germany, prepared locally) solution for 20 min, destained with 0.5% acid-alcohol for 2 min, counterstained with locally prepared 0.5% potassium permanganate (Riedel-deHaen, Germany) for 4 min, then air dried. The slide was rinsed with sterile water between each step. Then, bacilli examination was carried out using a light-emitting diode fluorescence microscopy (ZEISS Primo Star iLED fluorescence microscope, Germany) with a 40X objective [17,19].

#### Tissue processing, embedding, and sectioning

Punch biopsies in 10% formalin were kept for 48–72 h before tissue processing was performed overnight using an automated tissue processor (LEICA ASP 300S, Germany) as explained elsewhere [15]. The following day, the tissue was embedded in real-paraffin wax. A series of 4μm thick issue sections were prepared using a rotary microtome (LEICA RM2255, Germany) and fixed on one end of a frosted slide coated with 50% egg albumin–glycerol prepared locally. A total of three slides, each containing four consecutive sections from the same tissue were prepared for AO, FF and H&E staining [15].

#### H&E staining

One of the slides containing tissue sections was deparaffinized and rehydrated using a dry oven at 600 C for 30 min and in two changes of xylene for 10 min, in a series of decreasing concentrations of alcohol and finally in tap water. The slide was stained for 8 min in Harris’s hematoxylin reagent, destained in 0.5% acid-alcohol for 3 s and washed in running tap water. It was then counterstained with 0.5% eosin for 1 min. After dehydration with alcohol and xylene treatment, it was mounted with DPX mounting medium for histopathologic examination to be examined by a pathologist. Histopathologic features of leprosy like granulomas, epithelioid cells, foamy macrophages, giant cells, type of cell infiltration, and inflammation were used to diagnose and classify the disease into lepromatous leprosy (LL), borderline lepromatous (BL), borderline tuberculoid (BT), tuberculoid (TT), and indeterminate leprosy (INT) as described [15,29].

### FF and AO staining of tissue sections

Slides were warmed in an oven at 60°C for 10min and deparaffinized twice with two parts xylene and one part of vegetable oil, for 15 min, then blotted well with absorbent paper to remove the xylene-oil remnant and hydrated in a jar containing distilled water. For FF staining, the slide was flooded with filtered 1% calbolfuchsin for 20 minutes followed by destaining with 10% H_2_SO_4_ for 2 min. Tissue sections were then counter stained with 0.25% methylene blue solution for 20 s. The slide was rinsed with sterile water between each step. AO staining was performed as outlined above. Finally, for both staining procedures, after a final wash with water, slides were blotted, cleared with xylene, mounted with mounting medium (DPX mountant for histology, Sigma) and examined under the100X objective of the microscope or using LED-FM under the 40X objective, respectively [28,30,31].

### DNA extraction

DNA was extracted using three different methods. The first method called host depletion (HD) [32,33] removes host DNA and is therefore mainly used for whole-genome sequencing applications, but it can be also used for PCR application. HD was applied to 35 skin biopsies (S1 Table, S1 Fig). The second method used the QIAmp UCP Pathogen Mini kit (QiagenGmbH, Hilden, Germany) with an adapted protocol on 39 skin biopsies (S1 Table, S1 Fig). Briefly, biopsies were cut into small pieces in a 1.8mL micro-centrifuge tube. AHL Lysis buffer (500 μl) containing 20 μl of proteinase K (20mg/mL) was added to the disrupted tissue and incubated for 1h at 56°C. After mechanical lysis twice with 200 μl of 0.1 mm zirconium beads (Bertin Technology) at a velocity of 6.5m/s for 45 s with 5 min incubation on ice in a Precellys® 24 Instrument), a second round of enzymatic lysis was performed using 40 μl of proteinase K (20mg/mL) prior to incubation with APL2 buffer for 10min at 70°C. DNA was precipitated and purified on QIAamp UCP Pathogen Mini silica column followed by elution in 100 μl of elution buffer. In the third method, DNA was extracted from 91 skin biopsies (S1 Table, S1 Fig) using the QIAmp Fast DNA Tissue kit (QigenGmbH, Hilden, Germany) without mechanical lysis. Briefly, biopsies were cut into small pieces in a 1.8mL micro-centrifuge tube. AHL Lysis buffer (500 μl) containing 20 μl of proteinase K (20mg/mL) was added to the disrupted tissue and incubated 1h at 56°C, then for 10min at 70°C, as above. DNA was then precipitated, purified on QIAamp silica column and eluted in 100 μl of elution buffer.

### Polymerase chain reaction (PCR)

Conventional Polymerase Chain Reaction (PCR) was performed using primer pairs to detect the *M*. *leprae* specific repetitive region RLEP and the specific region in *hemN* from *M*. *Lepromatosis* in *M*. *leprae* PCR negative samples [32,34]. For each reaction, 3–5μl of extracted DNA was mixed with each primer (200nM final), 25μL of Accustart Master Mix and water in a final volume of 50μL. Amplification cycles started with a denaturation step at 95°C for 5min, followed by 40 cycles at 95°C for 30 s, annealing at 58°C for 40 s and extension at 72°C for 30 s. The reaction ended with an additional 10min extension step at 72°C. The amplified PCR product was then examined by agarose gel (1% w/v) electrophoresis.

### Quality control

Samples known to be positive or negative for *M*. *leprae* were used as positive and negative controls during the staining procedures and PCR.

### Data analysis

Based on previous experience of similar studies to develop a reference standard [35], we have established a combination of ZN, FF and H&E staining tests for this specific study. Clinical diagnosis was the necessary part of this combination supported by at least one or more positive test results of H&E, ZN and FF staining. This test panel was chosen due to their routine application in diagnosing leprosy in our laboratory and worldwide.

However, since the specificity of all these methods is known to be low, we will consider samples obtained from non-leprosy patients as truly negative for the specificity of AO staining in tissue, FF and PCR methods. A “true positive” will be a sample with one or more positive test results of H&E, ZN and FF staining. For ZN and AO in SSS, “true negatives” will be the negative samples obtained with the alternative gold standard method since SSS samples were not obtained from the non-leprosy patients (S1 and S2 Tables).

Socio-demographic data, clinical information and laboratory results were introduced into Stata SE version 11 for statistical analysis. Data obtained from four samples of leprosy cases were excluded from analysis due to incompleteness. Sensitivity, specificity, positive predictive value (PPV) and negative predictive value (NPV) were calculated including 95% confidence intervals (CI) against the designed alternative gold standard (S1 Fig). For statistical significance between the different detection methods, a binomial test (MacNemar test or the exact binomial test) and Fisher’s test were calculated in R when applied on the same group of samples and in case of independent groups, respectively (S1 Appendix).

Moreover, in other studies, patients commonly classified as TT, BT and INT are usually considered as PB patients with low BI whereas LL, BL and BB are classified as MB cases with high BI [36]. However, the WHO classification is based on the number of skin lesions and is not linked to the R&J classification because BI is either high or low. Moreover, it is also commonly accepted that INT,TT and BT samples are associated with low bacillary index whereas LL, BL and BB have higher bacillary index even if some exceptions can be observed. Thus, the MB and PB classification of this study is only relative to the number of skin lesions found per patient. Nevertheless, to compare the diagnostic performance of the methods described here with others published elsewhere, we have classified the patients based on the R&J classification as follows: TT, BT, INT and negative (NEG) will be considered as low BI samples and LL, BL and BB as high BI samples.

## Results

### Socio-demographic characteristics and clinical features

A total of 169 participants were involved in the study from January 2015 to April 2016 at ALERT center, Addis Ababa, Ethiopia. There were 141 leprosy cases and 28 in the non-leprosy control group (S1 and S2 Tables). Male study participants comprised 63.9% (108/169) with a male to female ratio of 1.7:1. The mean age and SD of study participants was 35. 8 ± 14.6 years with age ranging between 15 and 75 years.

### Clinical features

Among the clinically confirmed leprosy cases, 19.9% (28/141) showed ≤ 5 skin lesions and about 80.1% (113/141) presented with > 5 skin lesions and were classified accordingly as PB and MB (S1 Table). Visible physical disability was seen in 59.6% of the leprosy patients. Five (3.5%) participants who had completed MDT were categorized as relapse cases based on clinical criteria. A total of 32 (22.5%) participants presented with leprosy reactions classified as pure neuritis 15.6% (5/32), reversal reactions 68.8% (22/32) and 15.6% (5/32) with erythema nodusum leprosum. Regarding family history, 25.5% (36/141) of the leprosy cases used to live with a leprosy patient.

Among the non-leprosy control group, 32.1% (9/28) came to the hospital for surgical treatment of skin cancer, while the remaining 67.9% (19/28) came for different surgical treatment including corrective amputation but with no history of leprosy (S2 Table).

### The gold standard method

Among the 141 clinically confirmed leprosy cases, four samples were excluded from the analyses because of the absence of data for the microscopy methods (S1 Table, S1 Fig). A total of 137 clinically confirmed cases were analyzed and 113 were positive according to the gold standard method including 99 positive and 14 negative by histopathology (H&E) (Table 1, Table 2). All 28 samples from non-leprosy patients were negative using the gold standard method classification.

**Table 1:** Number and repartition of positive samples according to the gold standard method.

Histopathology (H&E)	Total	FF positive–ZN negative	FF negative–ZN positive	FF and ZN positive	FF and ZN negative
Positive	99	19	2	57	21
Negative	38	6	3	5	24

**Table 2:** Table: Histopathological repartition of the gold standard positive and negative samples with the number of positives and positivity rate (%) for each laboratory tests in each groups classified with the R&J classification–The table shows the high positivity rate for AO in punch biopsies and PCR compared to other methods.

	R&J classification	Number of patients per group	SSS		Punch biopsy		
			ZN (%)	AO (%)	FF (%)	AO (%)	PCR (%)
Gold standard positive	LL	25	22 (88)	24 (96)	25 (100)	25 (100)	25 (100)
	BL	20	19 (95)	19 (95)	20 (100)	20 (100)	20 (100)
	BB	14	8 (57.1)	9 (64.3)	10 (71.4)	10 (71.4)	13 (92.9)
	BT	25	7 (28)	12 (48)	14 (56)	17 (68)	23 (92)
	TT	9	0 (0)	2 (22.2)	4 (44.4)	5 (55.6)	5 (55.5)
	INT	6	3 (50)	3 (50)	4 (66.7)	3 (50)	5 (83.3)
	NEG	14	8 (57.1)	8 (57.1)	11 (78.6)	9 (64.3)	12 (85.7)
	Total	113	67 (59.3)	76 (67.3)	87 (77)	88 (77.9)	103 (91.1)
	LL, BL, BB	58	49(84.5)	51(87.9)	54(93.1)	54(93.1)	57(98.2)
	BT, TT, INT, NEG	55	18(32.7)	25(45.4)	33(60)	34(61.8)	46(83.6)
Gold standard negative		28	0 (0)	0 (0)	0 (0)	6 (25)	10 (41.7)

### Performance of leprosy diagnosis using Auramine O staining and PCR

#### Auramine O staining

On analyses of the 137 SSS, the sensitivity of AO in SSS (65.5%) was slightly higher (*p*>0.05) than ZN (59.3%) while specificity was 100% for both tests (Table 3, S3 Table).

**Table 3:** Diagnosis performance of the laboratory tests with 95% confidence interval (CI) based on the establish gold standard method–PCR_m+e:_ PCR result obtained on DNA extracted with mechanical (m) and enzymatic (e) methods; PCR_e_:: PCR result obtained on DNA extracted with enzymatic (e) method only.

Specimen type		Sensitivity		PPV		NPV	
		%	CI (95%)	%	CI (95%)	%	CI (95%)
SSS	ZN	59.3	49.6–68.4	100	94.6–100	34.3	23.3–46.6
	AO	65.5	56–74.2	100	95.1–100	38.1	26.1–51.2
Punch biopsy	H&E	87.6	80.1–93.1	100	96.3–100	66.7	50.5–80.4
	FF	77	68.1–84.4	100	95.8–100	51.8	37.8–65.7
	AO	77.9	69.1–85.1	100	95.9–100	52.8	38.6–66.7
	PCR	91.1	84.3–95.7	100	96.5–100	73.7	56.9–77.4
	PCR_m+e_	96.8	88.7–99.6	100	93.9–100	95	83.1–99.4
	PCR_e_	84.6	71.9–93.1	100	92–100	80	64.6–90.9

The sensitivity and specificity of 137 tissue sections stained with FF staining were 77% and 100%, respectively (Table 3), while other statistical parameters, PPV and NPV were 100% and 51.8%, respectively. Sensitivity and specificity of AO-tissue staining are similar (p>0.05) to FF with 77.9% and 100%, respectively, using the established gold standard method (Table 3, S3 Table).

The overall sensitivity of both AO in tissue and FF is significantly higher (*p*<0.05) than AO in SSS and ZN (S3 Table). In addition sensitivity of the different tests is higher in the form LL, BL and BB than TT, BT and INT (*p*<0.05) as expected since the number of bacilli is higher in the first three forms (Table 3, S3 Table).

### DNA extraction and PCR

PCR was positive for 103/113 gold standard positive leprosy (Table 2). DNA samples extracted from all of the non-leprosy control groups were negative as well as in 10 gold standard positive cases, probably because of the low BI (S1 Table). Since *M*. *lepromatosis* is also associated with leprosy cases in humans [37], all negative cases were also tested by PCR for the presence of *M*. *lepromatosis* as differential diagnosis for *M*. *leprae* infection. All 10 cases were PCR-negative (S1 Table).

The global sensitivity and specificity of the method were 91.1% and 100%, respectively (Table 3). Also, the performance of different DNA extraction methods (with or without mechanical lysis) was compared. For samples where DNA was extracted with mechanical lysis (HD and QiampUCP Pathogen kit, PCR_m+e_), the overall sensitivity is statistically higher (96.8%, *p*<0.05) compared to samples where DNA was extracted without mechanical lysis (Qiamp fast Pathogen, PCR_e_) with 86.7% (Table 3, S3 Table). The disparity between the two methods mainly occurs in the BT, TT, IND and NEG with a sensitivity of 95.5% with mechanical lysis and 76.7% without mechanical lysis (*p*>0.05) (Table 2, S3 Table).

### Diagnostic performance of laboratory test when leprosy cases are histopathologically classified and confirmed

**Fig 1:**
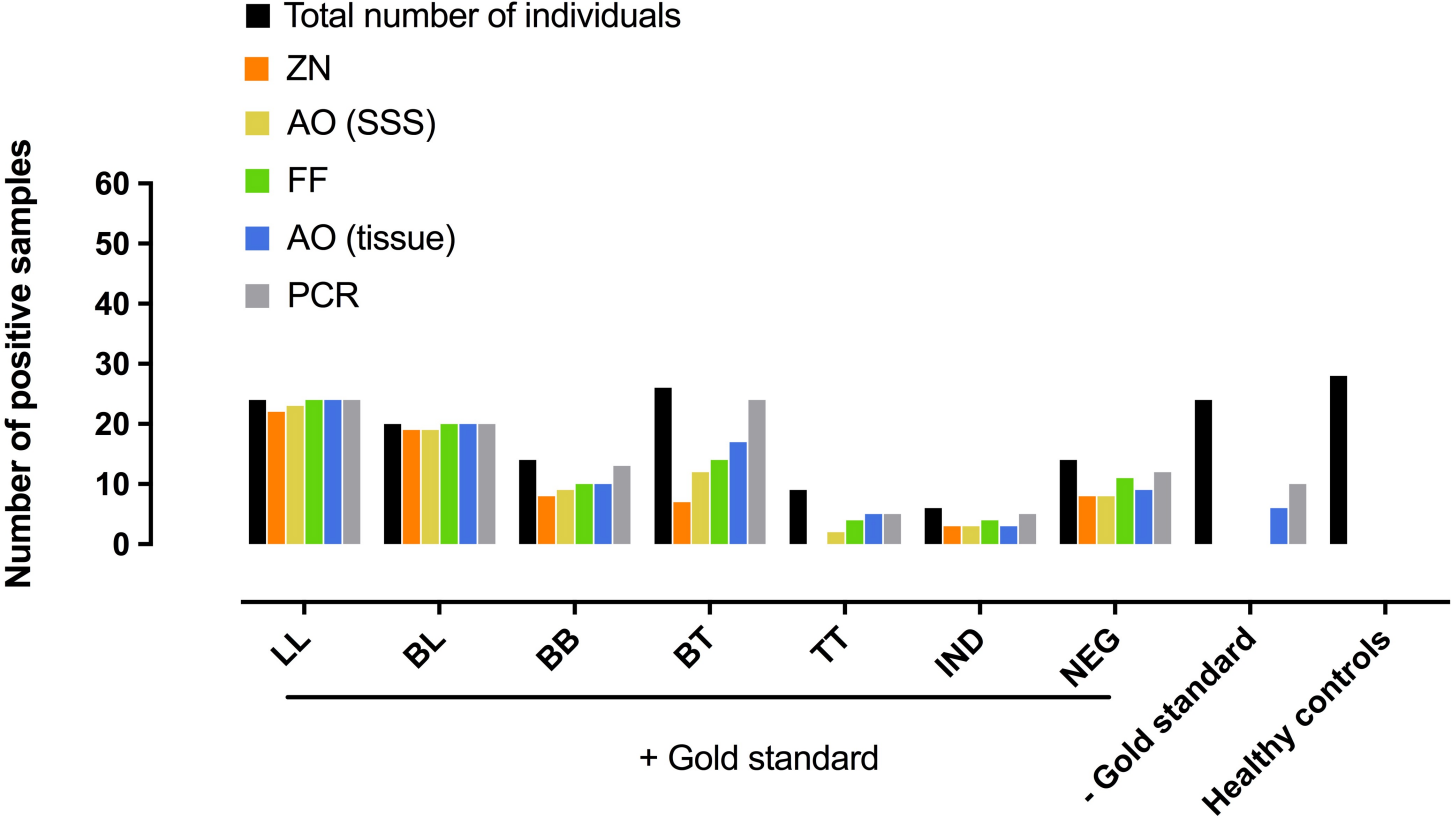
Histopathological repartition of the gold standard positive and negative samples with the number of positive sample and positivity rate (%) for each laboratory tests–HB: High bacillary load included samples from the LL, BL and BB groups; LB: Low bacillary load included samples from the BT, TT, INT and NEG groups–The graphic show the high positivity rate for AO-tissue and PCR in all groups compared to other methods.

Among the 58 (51.3%) histopathologically confirmed samples collectively classified under BB, BL or LL and expected to have high bacterial concentration, all laboratory tests gave similar results ranging from 84.5% to 98.3% with the highest sensitivity (*p*<0.05) recorded for PCR (Table 2, S3 Table). On the other hand, of the 55 (48.7%) samples classified as BT, TT, INT or Neg and thus expected to have few or no bacilli count (S1 Table), the sensitivity of AO in SSS (45.4%) is slightly higher than ZN (32.7%, *p*>0.05) whereas the sensitivity of AO in tissue (60%) is similar to FF (61.8%, *p*>0.05) but statistically lower than PCR (83.6%, *p*<0.05) (Fig 1, Table 3, S2 Fig and S1 Data).

### Negative cases

A total of 24 cases with clinical signs of leprosy were considered negative using the gold standard method. Regarding the number of lesions, 11 and 13 patients were classified as MB and PB, 17 presented with disabilities and nine reported a family history of leprosy (S2 Table). While ZN, FF and AO in SSS showed negative results, AO in tissue and PCR were positive for six and ten cases, respectively, including four positive samples common to both methods (Fig 1, S3 Table).

## Discussion

Current leprosy diagnosis relies upon clinical examination of the patient, recognition of skin lesions and peripheral neuropathy, in addition to identification of acid fast bacilli and histopathology typical of the active lesion. However, the identification of a true leprosy case when disabilities are not yet visible, especially for PB patients, is a challenge for clinician [38,39]. Therefore, histopathology is still mostly used as the gold standard for the diagnosis, with some limitations [25]. This method is less specific compared to the mycobacterial staining methods, but different reports describe a valuable sensitivity of histopathologic analysis for some doubtful cases [40–42]. In this study, 72% (99/137) of the clinically identified leprosy patients would have been considered positive based solely on H&E staining and clinical signs (Table 1). Using a combination of methods, as suggested by Reja*et al*, including in this case the H&E staining and FF on tissue with ZN on SSS, increased the number of positives to 82% (113/137) [25].

**Fig 2:**
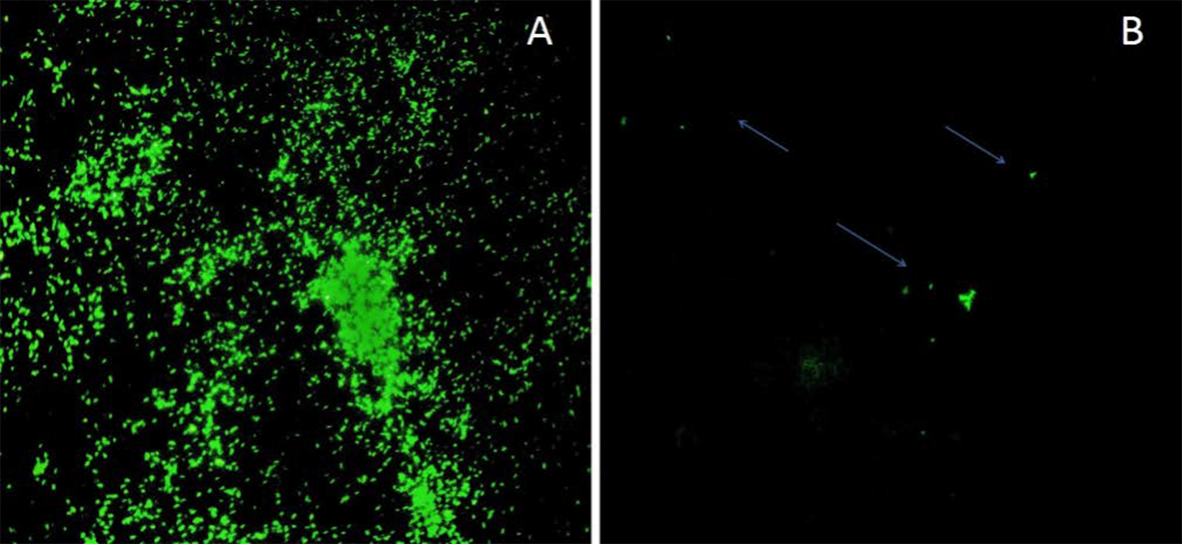
Auramine O stained *M*.*leprae* in FFPE tissue section under 40X objective of light-emitting diode fluorescence microscope A: Sample with high BI B. Sample with low BI.

ZN is uncomplicated, cost-effective and the most frequently used method for the detection of AFB especially in resource limited settings. The sensitivity of ZN is inconsistent ranging from 18% to 56%, depending on the study [23–25,43].We reported a sensitivity of 59.3% respectively, with a low negative predictive value demonstrating the probable high rate of false negative for ZN. An acceptable alternative would be AO staining on SSS with a slightly higher percentage of positivity (64.9%, *p*>0.05). While the difference is not significant, AO staining is simpler due to the ease of detection of fluorescently stained bacilli and the ability to screen the entire field within a short period (Fig 2).

FF staining is another widely-accepted laboratory diagnostic test for leprosy on tissue sections. Though its specificity is usually high as suggested by our results and others [20], the sensitivity of FF is affected by the type of disease, as are most of the other laboratory tests for leprosy. Nayak et al. reported an FF sensitivity of 44.6% and 60%, respectively for PB and MB patients, whereas we report 56.9% and 93.1% for BT, TT, IND, NEG and LL, BL and BB cases, respectively [20]. The ALERT hospital is specialized in diagnosis of dermatological diseases and has many senior dermatologists. In this study, the difference between the sensitivity value is most probably linked to the definition of the gold standard method and the involvement of highly skilled dermatologists. Here, FF and AO in tissue are more sensitive than ZN (*p*<0.05) and the global sensitivity between FF (77%) and AO (77.9%) in tissue are similar. The detection rate obtained for AO staining is similar to that in previously published studies [19]. Moreover, sensitivity is identical between both methods for all classification forms suggesting that AO staining on the tissue (Fig 2) could replace FF without any loss of sensitivity. In addition, the sensitivity of both FF and AO in tissue section is higher (*p*<0.05) compared to SSS for LB cases. This suggests that tissue sections should be preferred to SSS for leprosy diagnosis.

PCR is often acknowledged for its great sensitivity among all laboratory diagnostic tests [22,23]. A study in Brazil reported PCR sensitivity of 40% for TT, 55.5% for BT and 100% for all BB, BL and LL cases, respectively [44]. The authors concluded that PCR improves the diagnostic efficiency of low BI cases which mostly have a negative BI [44]. In our study, the result for LL, BL and BB samples was comparable with that of the Brazilian study but the sensitivity found for the BT, TT and INT cases with 92%, 55.5% and 83.3% respectively was relatively higher. Even though we were not able to confirm independently the histopathologic classification, we emphasize that the possible reason for this higher sensitivity is the use of a more effective DNA extraction method. *M*. *leprae* is an intracellular pathogen with an elaborate cell wall which confers resistance to alcohol and acid treatment as well as to standard pathogen lysis methods. Altogether, these characteristics should be taken into consideration to ensure proper DNA recovery. The importance of the extraction method used to obtain *M*. *leprae* DNA is often underestimated. Indeed, in this study, we detected more positive cases when chemical lysis was combined with mechanical lysis during DNA extraction with an increase of sensitivity (p>0.05) from 84.6% to 96.8% compared to DNA extraction using chemical lysis alone (Table 2).

Finally, 10/24 PCR samples among the negative cases, classified by the gold standard method established here, were positive by PCR for which specificity was 100% in our investigation and previous [45]. In previous studies, false positives have been observed in samples from patients with other skin diseases but this was probably due to misdiagnosis in the first place [44]. To avoid false positives, only patients with no family history of leprosy were included in the non leprosy control group and all skin samples were analyzed with standard methods such as H&E and FF. Thus, the rate of positivity in the negative gold standard group is highly encouraging to recommend PCR even for routine diagnosis. Overall, these results indicate the potential value of a single run of PCR to support clinical diagnosis rapidly without the requirement of pathologists and the other staining tests included in the alternatively establish the gold standard method in the study. However, drawback of conventional endpoint PCR is non-quantitative nature. Currently, several quantitative PCR tests have been optimized for detection of *M*. *leprae* but the cost and the absence of a standardized protocol is a limitation to its implementation at lower level health institutions in resource-limited countries [44,46,47].

## Supporting information

S1 Checklist STARD checklist. (DOCX)

S1 Data Commands and output data for the statistical analysis in R. (DOCX)

S1 STARD Flow diagram for AO staining in slit skin smear. (PDF)

S2 STARD Flow diagram for AO staining in skin biopsies. (PDF)

S3 STARD Flow diagram for PCR. (PDF)

S4 STARD Flow diagram for Fite faraco staining. (PDF)

S5 STARD Flow diagram for Zhiel Neelsen staining. (PDF)

S1 Table Overview of the socio-demographic and clinical characteristic of leprosy cases included in this study with results of the laboratory tests–RR: Reversal reaction, ENL: Erythema Nosodum, N: Neuritis–NA: Non available; ND: Not done. (XLSX)

S2 Table Overview of the socio-demographic and clinical characteristic of non-leprosy cases included in this study with results of the laboratory tests and other diseases associated. (XLSX)

S3 Table Statistical value obtained with the binomial tests and the Fisher test for the different tests in different condition (all samples, LB = low bacillary (BT, TT and INT) or HB = high bacillary (LL, BL and BB))–*p* = *p-value* in red when *p*> 0.05 and in green when *p*<0.05; OR: odds ratio.
(DOCX)

S1 Fig Flowchart of the study design representing the collection of data and samples in addition to the number of sample collected and the methods applied to these samples–HD: Host depletion, QIAmp UCP: QIAmp UCP Pathogen Mini kit, QIAmp fast: QIAmp Fast DNA Tissue kit, m+e (red): Mechanical and enzymatic digestion, e (blue): Enzymatique digestion only. (DOCX)

S2 Fig Raw data used for the calculation of the sensitivity, specificity, PPV and NPV of the routine methods, AO and PCR- Each value represented the number of patients per group. (DOCX)
