# Supplementary material for: Evaluation of Auramine O staining and conventional PCR for leprosy diagnosis: A comparative cross-sectional study from Ethiopia
Source: PLoS Negl Trop Dis. 2018 Sep 4;12(9):e0006706. doi: 10.1371/journal.pntd.0006706 (PMC6138420; doi:10.1371/journal.pntd.0006706)
Supplement: S1 Data — (DOCX) [file pntd.0006706.s002.docx]

**Supplementary materials for**

**Evaluation of auramine O Staining and conventional PCR for leprosy diagnosis: a comparative cross-sectional study from Ethiopia**

**Short Title: Auramine O staining and PCR for leprosy diagnosis**

Authors: Selfu Girma^1*^, Charlotte Avanzi^2^, Kidist Bobosha^1*^, Kassu Desta^2^, Munir H. Idris^1^, Philippe Busso^2^, Yohannes Tsegaye^1^, Shimelis Nigusse^4^, Tsegaye Hailu^1^, Stewart T. Cole^2, 5^, Abraham Aseffa^1^.

^1^Armauer Hansen Research Institute, Ethiopia

^2^ Global Health Institute, École Polytechnique Fédérale de Lausanne, Switzerland

^3^Addis Ababa University, CHS, Department of Medical Laboratory Sciences, Ethiopia

^4^ ALERT hospital, Ethiopia

^5^ Institut Pasteur de Paris, France

* Corresponding authors

Selfu Girma: [selfugirma2@gmail.com](mailto:selfugirma2@gmail.com)

Kidist Bobosha: [kbobosha@gmail.com](mailto:kbobosha@gmail.com)

S1 Table: Overview of the socio-demographic and clinical characteristic of leprosy cases included in this study with results of the laboratory tests – RR: reversal reaction, ENL: erythema Nosodum, N: neuritis

S2 Table: Overview of the socio-demographic and clinical characteristic of non-leprosy cases included in this study with results of the laboratory tests and other diseases associated

S3 Table: Statistical value obtained with the binomial tests and the Fisher test for the different tests in different condition (all samples, LB=low bacillary (BT, TT and INT) or HB=high bacillary(LL, BL and BB)) – p= p-value in red when p> 0.05 and in green when p<0.05; OR: odds ratio;

S1 Fig: Raw data used for the calculation of the sensitivity, specificity, PPV and NPV of the routine methods, AO and PCR- Each value represented the number of patients per group

S2 Fig: Flowchart of the study design representing the collection of data and samples in addition to the number of sample collected and the methods applied to these samples – HD: host depletion, QIAmp UCP: QIAmp UCP Pathogen Mini kit, QIAmp fast: QIAmp Fast DNA Tissue kit, m+e (red): mechanical and enzymatic digestion, e (blue): enzymatique digestion only

S1 Appendix: Commands and output data for the statistical analysis in R

- Exact binomial test (number of discordant <20) or Mac Nemar test with continuity correction (number of discordant >20)

**For the test in SSS: ZN *vs.* AO: not significative**

> ZNvsAO= as.table(rbind(c(61, 10), c( 3, 36) ))

> colnames(ZNvsAO) <- rownames(ZNvsAO) <- c("pos", "neg")

> names(dimnames(ZNvsAO)) = c("AO", "ZN")

> ZNvsAO

> addmargins(ZNvsAO)

> ZNvsAO_1 <- addmargins(ZNvsAO, FUN = list(Total = sum), quiet = TRUE)

> #add margin in row and column

> n12<- ZNvsAO_1[1,2]

> n21<- ZNvsAO_1[2,1]

> ns<-n12+n21

> if(ns>20) {cat("ns = ", ns, ". The fonction mcnemar.test() sould be used \n", sep="")} else {cat("ns = ", ns, ". The fonction binom.test() should be used \n", sep="")}

ns = 13. The fonction binom.test() should be used

> binom.test(n12,ns,0.5)

Exact binomial test

data: n12 and ns

number of successes = 10, number of trials = 13, ***p-value* = 0.09229**

alternative hypothesis: true probability of success is not equal to 0.5

95 percent confidence interval: [0.4618685 0.9496189]

sample estimates: probability of success = 0.7692308

**For the test in punch biopsy: FF *vs.* AO: not significative**

> FFvsAO= as.table(rbind(c(83, 5), c( 4, 21) ))

> colnames(FFvsAO) <- rownames(FFvsAO) <- c("pos", "neg")

> names(dimnames(FFvsAO)) = c("AO", "FF")

> FFvsAO

> addmargins(FFvsAO)

> FFvsAO_1 <- addmargins(FFvsAO, FUN = list(Total = sum), quiet = TRUE)

> #add margin in row and column

> n12<- FFvsAO_1[1,2]

> n21<- FFvsAO_1[2,1]

> ns<-n12+n21

> if(ns>20) {cat("ns = ", ns, ". The fonction mcnemar.test() sould be used \n", sep="")} else {cat("ns = ", ns, ". The fonction binom.test() should be used \n", sep="")}

ns = 9. The fonction binom.test() should be used

> binom.test(n12,ns,0.5)

Exact binomial test

data: n12 and ns

number of successes = 5, number of trials = 9, ***p-value* = 1**

alternative hypothesis: true probability of success is not equal to 0.5

95 percent confidence interval: [0.2120085-0.8630043]

sample estimates: probability of success = 0.5555556

**For the test in punch biopsy: FF *vs.* PCR: significative**

> FFvsPCR= as.table(rbind(c(81, 22), c( 6, 4) ))

> colnames(FFvsPCR) <- rownames(FFvsPCR) <- c("pos", "neg")

> names(dimnames(FFvsPCR)) = c("PCR", "FF")

> FFvsPCR

> addmargins(FFvsPCR)

> FFvsPCR_1 <- addmargins(FFvsPCR, FUN = list(Total = sum), quiet = TRUE)

> #add margin in row and column

> n12<- FFvsPCR_1[1,2]

> n21<- FFvsPCR_1[2,1]

> ns<-n12+n21

> if(ns>20) {cat("ns = ", ns, ". The fonction mcnemar.test() sould be used \n", sep="")} else {cat("ns = ", ns, ". The fonction binom.test() should be used \n", sep="")}

ns = 28. The fonction mcnemar.test() sould be used

> mcnemar.test(FFvsPCR_1,correct = TRUE)

McNemar's Chi-squared test

data: FFvsPCR_1

McNemar's chi-squared = 17.601, df = 3, ***p-value* = 0.0005315**

**For the test in punch biopsy: PCR *vs.* AO: significative**

> PCRvsAO= as.table(rbind(c(84, 4), c( 19, 4) ))

> colnames(PCRvsAO) <- rownames(PCRvsAO) <- c("pos", "neg")

> names(dimnames(PCRvsAO)) = c("AO", "PCR")

> PCRvsAO

> addmargins(PCRvsAO)

> PCRvsAO_1 <- addmargins(PCRvsAO, FUN = list(Total = sum), quiet = TRUE)

> #add margin in row and column

> n12<- PCRvsAO_1[1,2]

> n21<- PCRvsAO_1[2,1]

> ns<-n12+n21

> if(ns>20) {cat("ns = ", ns, ". The fonction mcnemar.test() sould be used \n", sep="")} else {cat("ns = ", ns, ". The fonction binom.test() should be used \n", sep="")}

ns = 23. The fonction mcnemar.test() sould be used

>

>

> mcnemar.test(PCRvsAO_1,correct = TRUE)

McNemar's Chi-squared test

data: PCRvsAO_1

McNemar's chi-squared = 18.219, df = 3, ***p-value* = 0.0003965**

**For the test in SSS: ZN *vs.* AO in LB samples: not significative**

> ZNvsAO_LB= as.table(rbind(c(16, 7), c( 2, 30)))

> colnames(ZNvsAO_LB) <- rownames(ZNvsAO_LB) <- c("pos", "neg")

> names(dimnames(ZNvsAO_LB)) = c("AO", "ZN")

> ZNvsAO_LB

> addmargins(ZNvsAO_LB)

> ZNvsAO_LB_1 <- addmargins(ZNvsAO_LB, FUN = list(Total = sum), quiet = TRUE)

> #add margin in row and column

> n12<- ZNvsAO_LB_1[1,2]

> n21<- ZNvsAO_LB_1[2,1]

> ns<-n12+n21

> if(ns>20) {cat("ns = ", ns, ". The fonction mcnemar.test() sould be used \n", sep="")} else {cat("ns = ", ns, ". The fonction binom.test() should be used \n", sep="")}

ns = 13. The fonction binom.test() should be used

> binom.test(n12,ns,0.5)

Exact binomial test

data: n12 and ns

number of successes = 7, number of trials = 9, ***p-value* = 0.1797**

alternative hypothesis: true probability of success is not equal to 0.5

95 percent confidence interval: [0.3999064-0.9718550]

sample estimates: probability of success = 0.7692308

**For the test in punch biopsy: FF *vs.* AO in LB samples: not significative**

> FFvsAO_LB= as.table(rbind(c(29, 5), c( 4, 17)))

> colnames(FFvsAO_LB) <- rownames(FFvsAO_LB) <- c("pos", "neg")

> names(dimnames(FFvsAO_LB)) = c("AO", "FF")

> addmargins(FFvsAO_LB)

> FFvsAO_LB_1 <- addmargins(FFvsAO_LB, FUN = list(Total = sum), quiet = TRUE)

> n12<- FFvsAO_LB_1[1,2]

> n21<- FFvsAO_LB_1[2,1]

> ns<-n12+n21

> if(ns>20) {cat("ns = ", ns, ". The fonction mcnemar.test() sould be used \n", sep="")} else {cat("ns = ", ns, ". The fonction binom.test() should be used \n", sep="")}

ns = 9. The fonction binom.test() should be used

> binom.test(n12,ns,0.5)

Exact binomial test

data: n12 and ns

number of successes = 5, number of trials = 9, ***p-value* = 1**

alternative hypothesis: true probability of success is not equal to 0.5

95 percent confidence interval: [0.2120085-0.8630043]

sample estimates: probability of success = 0.5555556

**For the test in punch biopsy: FF *vs.* PCR in LB samples: significative**

> FFvsPCR_LB= as.table(rbind(c(27, 19), c( 6, 3) ))

> colnames(FFvsPCR_LB) <- rownames(FFvsPCR_LB) <- c("pos", "neg")

> names(dimnames(FFvsPCR_LB)) = c("PCR", "FF")

> addmargins(FFvsPCR_LB)

> FFvsPCR_LB_1 <- addmargins(FFvsPCR_LB, FUN = list(Total = sum), quiet = TRUE)

> n12<- FFvsPCR_LB_1[1,2]

> n21<- FFvsPCR_LB_1[2,1]

> ns<-n12+n21

> if(ns>20) {cat("ns = ", ns, ". The fonction mcnemar.test() sould be used \n", sep="")} else {cat("ns = ", ns, ". The fonction binom.test() should be used \n", sep="")}

ns = 28. The fonction mcnemar.test() sould be used

>

>

> binom.test(n12,ns,0.5)

Exact binomial test

data: n12 and ns

number of successes = 19, number of trials = 25, ***p-value* = 0.01463**

alternative hypothesis: true probability of success is not equal to 0.5

95 percent confidence interval: [0.5487120-0.9064356394]

sample estimates: probability of success = 0.76

**For the test in punch biopsy: PCR *vs.* AO_LB samples: significative**

> PCRvsAO_LB= as.table(rbind(c(30,4), c( 16,5) ))

> colnames(PCRvsAO_LB) <- rownames(PCRvsAO_LB) <- c("pos", "neg")

> names(dimnames(PCRvsAO_LB)) = c("AO", "PCR")

> addmargins(PCRvsAO_LB)

> PCRvsAO_LB_1 <- addmargins(PCRvsAO_LB, FUN = list(Total = sum), quiet = TRUE)

> n12<- PCRvsAO_LB_1[1,2]

> n21<- PCRvsAO_LB_1[2,1]

> ns<-n12+n21

> if(ns>20) {cat("ns = ", ns, ". The fonction mcnemar.test() sould be used \n", sep="")} else {cat("ns = ", ns, ". The fonction binom.test() should be used \n", sep="")}

ns = 13. The fonction binom.test() should be used

> binom.test(n12,ns,0.5)

Exact binomial test

data: n12 and ns

number of successes = 4, number of trials = 20, ***p-value* = 0.01182**

alternative hypothesis: true probability of success is not equal to 0.5

95 percent confidence interval: [0.057334-0.436614]

sample estimates: probability of success = 0.2

- Fisher test

**Comparison of the DNA extraction method: PCR_m+c_ vs PCR_c_ (significative)**

Fisher's Exact Test for Count Data

data: matrix(c(59, 44, 2, 8), nrow = 2)

***p-value* = 0.04203**

95 percent confidence interval: [0.9885374-53.5121921]

sample estimates: **odds ratio = 5.289797**

**Comparison of the DNA extraction method in LB group: PCR_m+c_ vs PCR_c_ (non significative)**

Fisher's Exact Test for Count Data

data: matrix(c(23, 2, 23, 7), nrow = 2)

***p-value* = 0.1596**

95 percent confidence interval: [0.3295822 -196.5993184]

sample estimates: **odds ratio = 3.712408**

**Comparison of ZN in HBvsLB SSS samples (significative)**

Fisher's Exact Test for Count Data

data: matrix(c(49, 9, 18, 37), ncol=2)

***p-value* = 1.691e-08**

95 percent confidence interval: [4.183375-31.140705]

sample estimates: **odds ratio = 10.89855**

**Comparison of FF in HBvsLB in in punch biopsy samples (significative)**

Fisher's Exact Test for Count Data

data: matrix(c(54, 4, 33, 22), ncol=2)

***p-value* = 3.294e-05**

95 percent confidence interval: [2.675843 38.348200]

sample estimates: **odds ratio = 8.826241**

**Comparison of AO in HBvsLB SSS samples (significative)**

Fisher's Exact Test for Count Data

data: matrix(c(51, 7, 23, 32), ncol=2)

***p-value* = 3.543e-07**

95 percent confidence interval: [3.627156-30.682139]

sample estimates: **odds ratio = 9.900682**

**Comparison of AO in HBvsLB in punch biopsy (significative)**

Fisher's Exact Test for Count Data

data: matrix(c(34, 21, 54, 4), ncol=2)

***p-value* = 7.244e-05**

alternative hypothesis: true odds ratio is not equal to 1

95 percent confidence interval: [2.471601-35.601641]

sample estimates: **odds ratio = 8.184024**

**Comparison of PCR in HBvsLB in punch biopsies (significative)**

Fisher's Exact Test for Count Data

data: matrix(c(57, 1, 46, 9), ncol=2)

***p-value* = 0.007251**

95 percent confidence interval: [1.429318-495.967339]

sample estimates: **odds ratio = 10.96275**

**Comparison of FF *vs* ZN (significative)**

Fisher's Exact Test for Count Data

data: matrix(c(87,26, 67, 46), ncol=2)

***p-value* = 0.006469**

95 percent confidence interval: [1.243370-4.279361]

sample estimates: **odds ratio = 2.288787**

**Comparison of FF *vs.* AO in SSS (not significative)**

Fisher's Exact Test for Count Data

data: matrix(c(87,26, 74, 39), ncol=2)

***p-value* = 0.07739**

95 percent confidence interval: [0.9448092 3.3151129]

sample estimates: **odds ratio = 1.759098**

**Comparison of AO in tissue *vs.* ZN (significative)**

Fisher's Exact Test for Count Data

data: matrix(c(88, 25, 67, 46), ncol=2)

***p-value* = 0.003991**

95 percent confidence interval: [1.301635 4.528584]

sample estimates: **odds ratio = 2.407134**

**Comparison of AO in tissue *vs.* AO in SSS (significative)**

Fisher's Exact Test for Count Data

data: matrix(c(88, 25, 74, 39), ncol=2)

***p-value* = 0.05448**

95 percent confidence interval: [0.9891871 3.5077628]

sample estimates: **odds ratio = 1.850064**

**Comparison of PCR *vs.* FF (significative)**

Fisher's Exact Test for Count Data

data: matrix(c(103, 10, 87, 26), ncol=2)

***p-value* = 0.005828**

95 percent confidence interval: [1.339432-7.533142]

sample estimates: **odds ratio = 3.063249**

**Comparison of PCR *vs.* AO in SSS (significative)**

Fisher's Exact Test for Count Data

data: matrix(c(103, 10, 88, 25), ncol=2)

***p-value* = 0.009314**

95 percent confidence interval: [1.26725-7.18700]

sample estimates: **odds ratio = 2.912591**

**Comparison of AO in tissue *vs.* AO in SSS in LB (significative)**

Fisher's Exact Test for Count Data

data: matrix(c(23, 32, 34, 21), ncol=2)

***p-value* = 0.05588**

95 percent confidence interval: [0.9820084 5.1914399]

sample estimates: **odds ratio = 2.235567**

**Comparison of FF *vs.* AO in SSS in LB (not significative)**

Fisher's Exact Test for Count Data

data=matrix(c(33, 22 ,23,32), ncol=2)

***p-value* = 0.08562**

95 percent confidence interval: [0.9129678-4.7905637]

sample estimates: **odds ratio = 2.072738**

**Comparison of PCR *vs.* AO in SSS in LB (significative)**

Fisher's Exact Test for Count Data

data=matrix(c(46,9,23,32), ncol=2)

***p-value* = 9.831e-06**

95 percent confidence interval: [2.705037-19.565706]

sample estimates: **odds ratio = 6.970449**

**Comparison of PCR *vs.* ZN in LB (significative)**

Fisher's Exact Test for Count Data

data=matrix(c(46,9,18, 37), ncol=2)

***p-value* = 8.842e-08**

95 percent confidence interval: [3.913352-29.354623]

sample estimates: **odds ratio = 10.23571**

**Comparison of AO in tissue *vs.* ZN in LB samples: significative**

Fisher's Exact Test for Count Data

data=matrix(c(34, 21, 18, 37), ncol=2)

***p-value* = 0.003967**

95 percent confidence interval: [1.420297-7.858316]

sample estimates: **odds ratio = 3.289607**

**Comparison of FF *vs.* ZN in LB samples: significative**

Fisher's Exact Test for Count Data

data=matrix(c(33, 22, 18, 37), ncol=2)

***p-value* = 0.007151**

95 percent confidence interval: [1.320439-7.253882]

sample estimates: **odds ratio = 3.050276**
