# Supplementary figures and images for: Evaluation of Auramine O staining and conventional PCR for leprosy diagnosis: A comparative cross-sectional study from Ethiopia
Source: PLoS Negl Trop Dis. 2018 Sep 4;12(9):e0006706. doi: 10.1371/journal.pntd.0006706 (PMC6138420; doi:10.1371/journal.pntd.0006706)

Prototypical STARD diagram to report flow of participants through the study

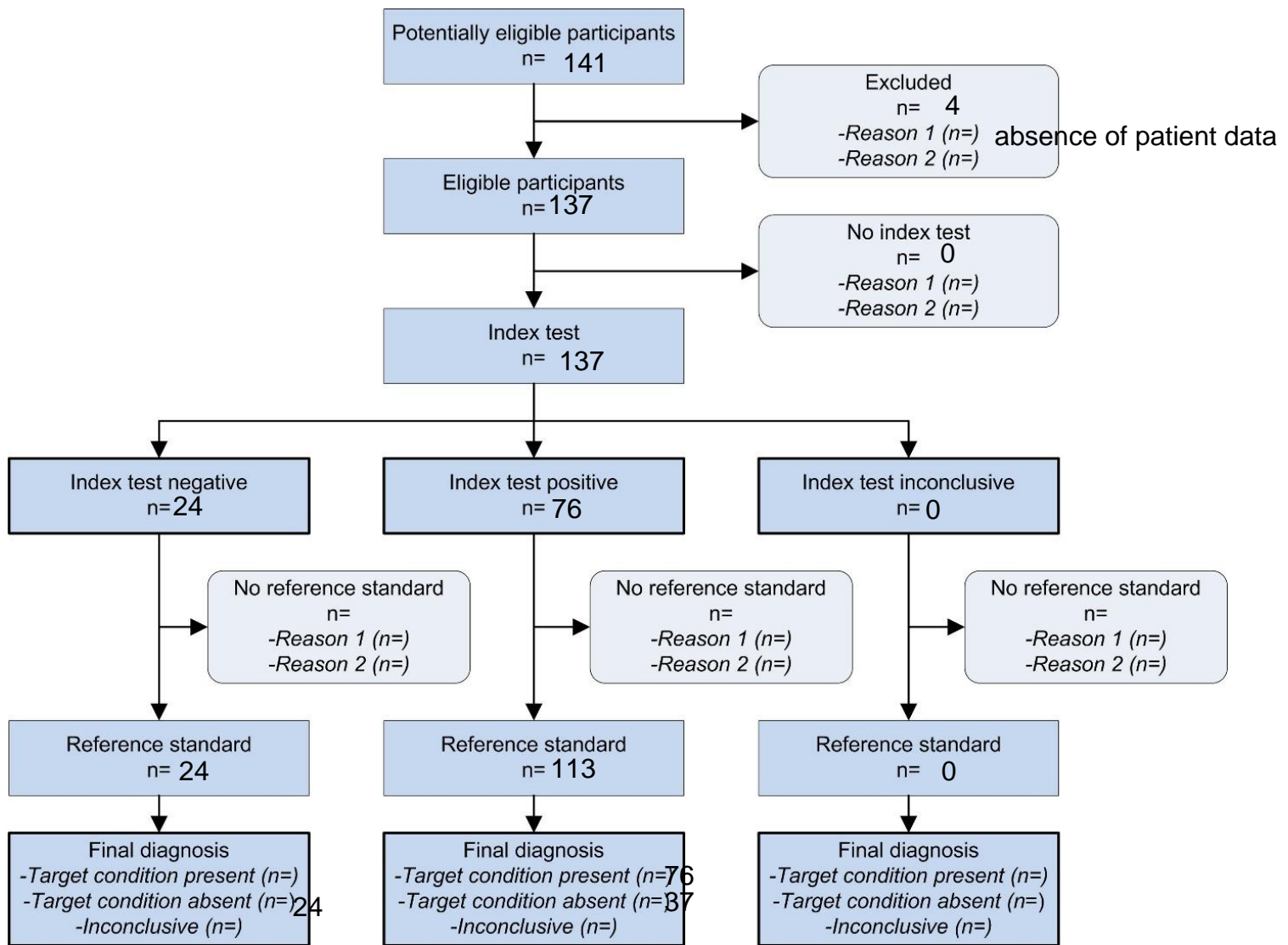

Supplement: S1 STARD — (PDF) [file pntd.0006706.s003.pdf]

Prototypical STARD diagram to report flow of participants through the study

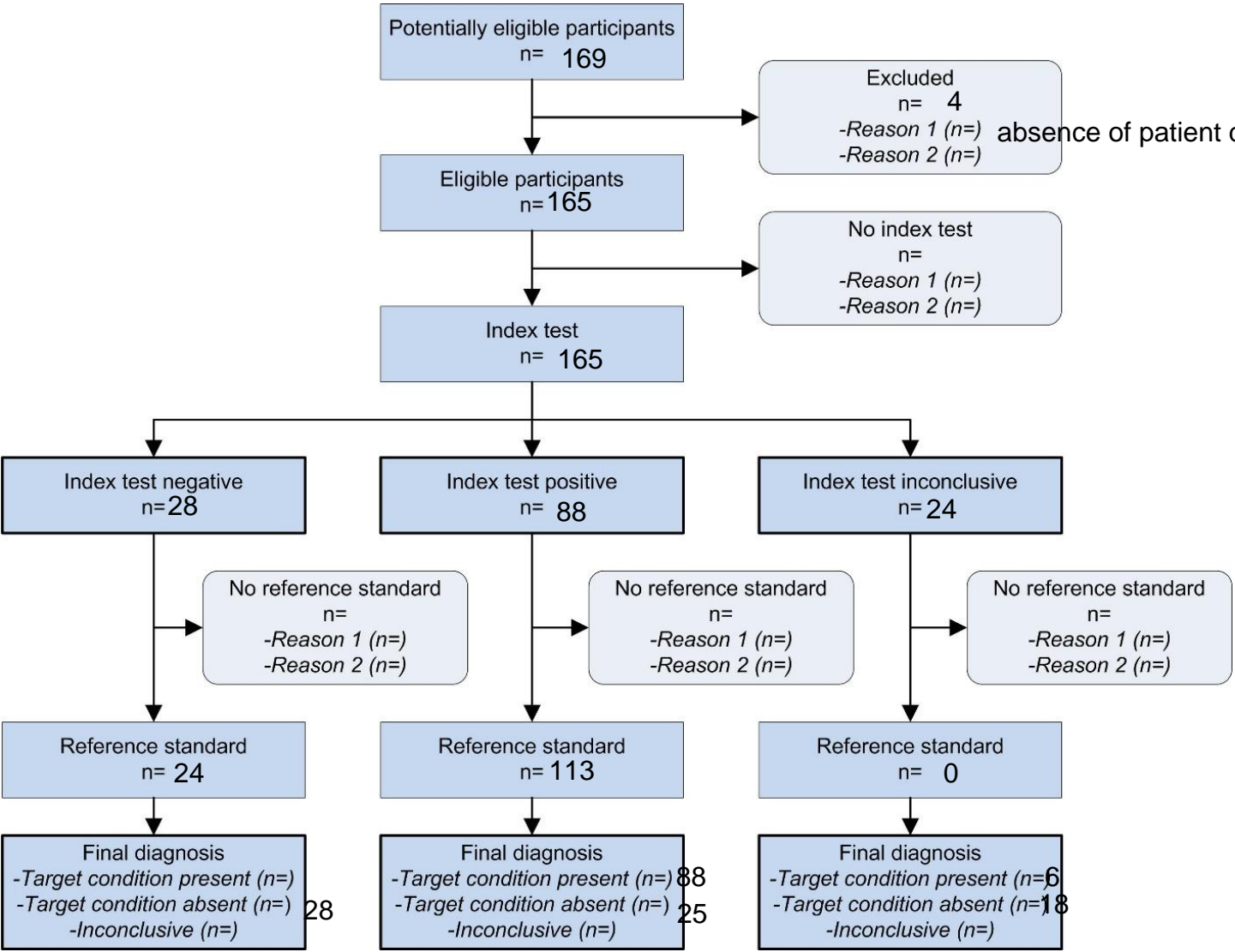

Supplement: S2 STARD — (PDF) [file pntd.0006706.s004.pdf]

Prototypical STARD diagram to report flow of participants through the study

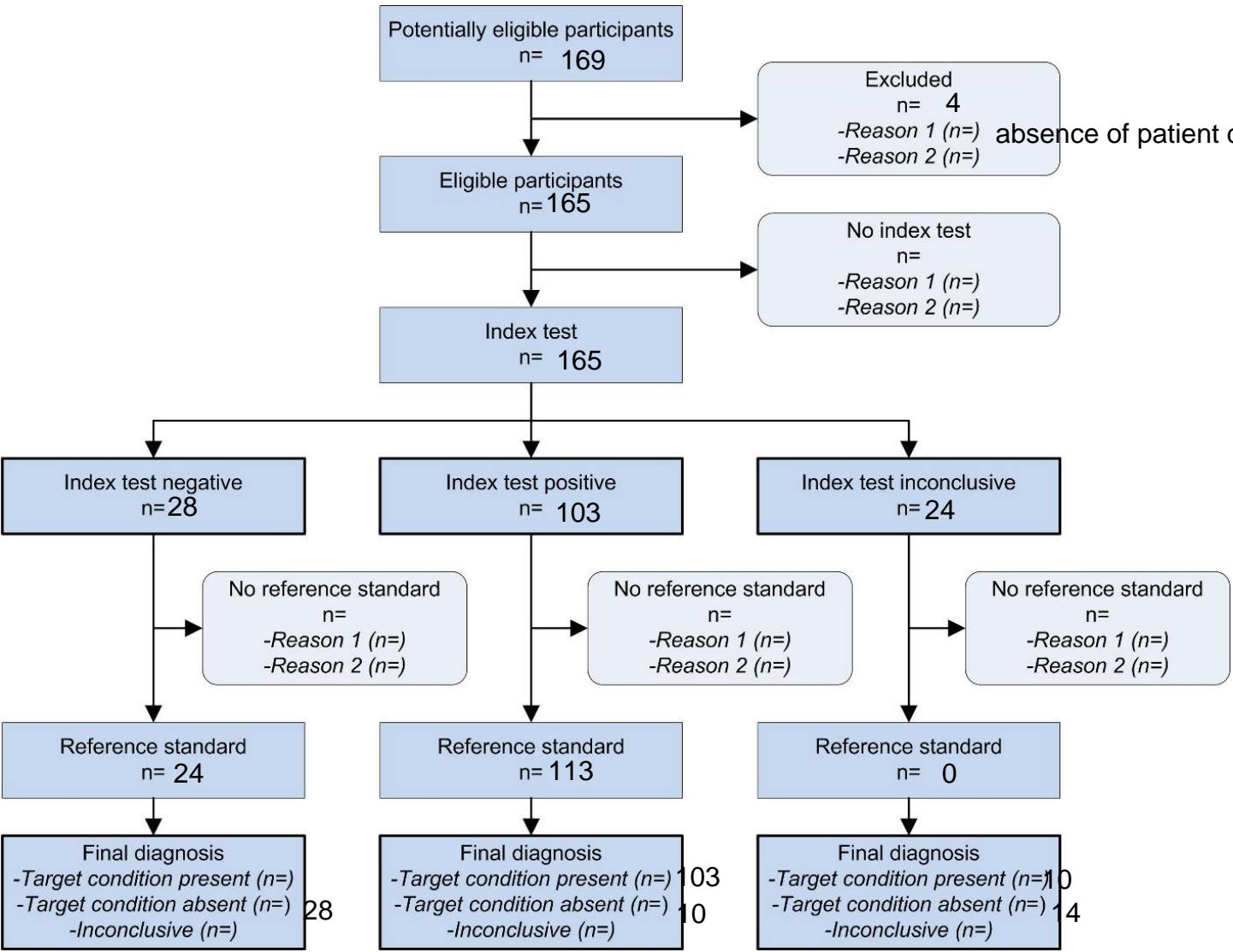

Supplement: S3 STARD — (PDF) [file pntd.0006706.s005.pdf]

Prototypical STARD diagram to report flow of participants through the study

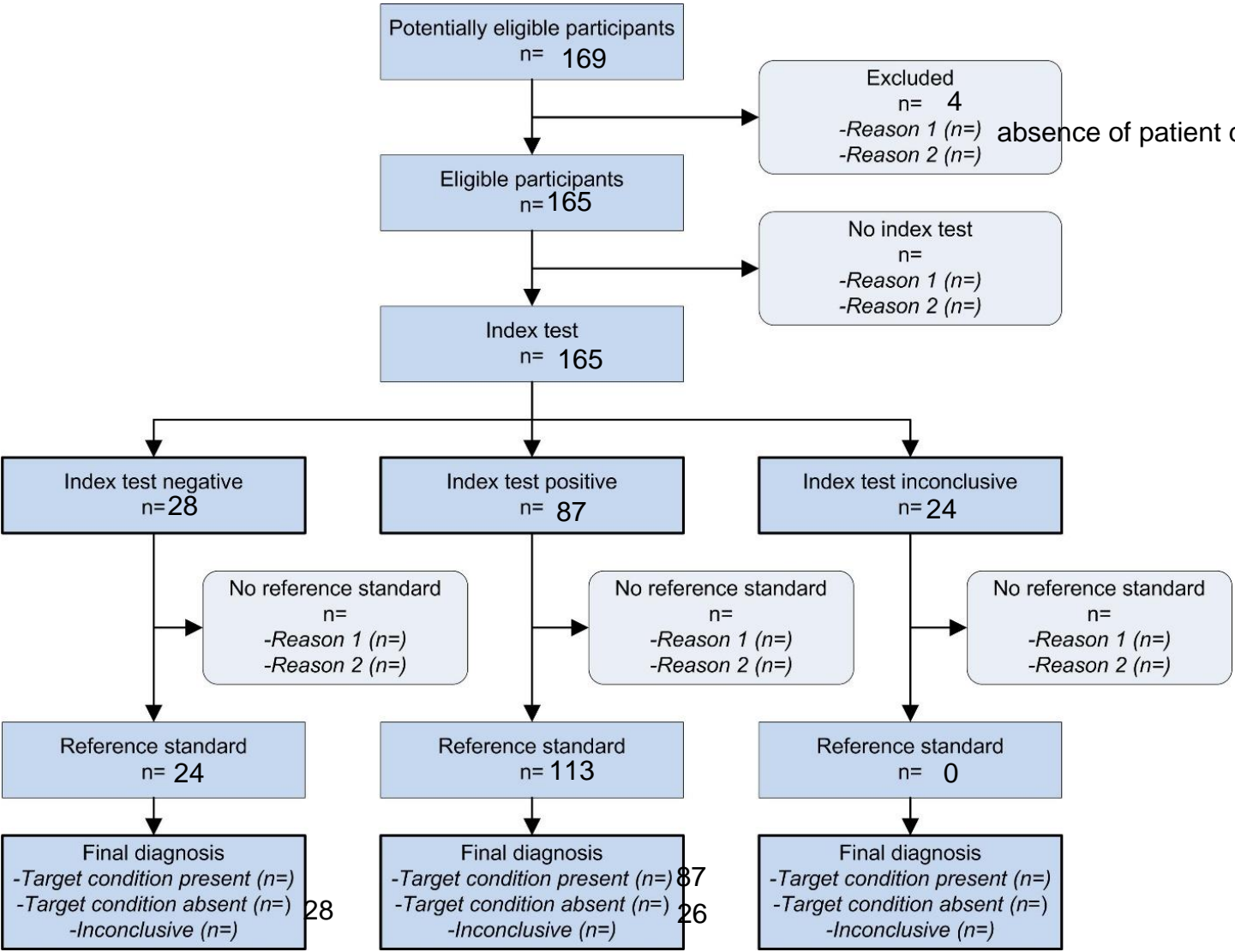

Supplement: S4 STARD — (PDF) [file pntd.0006706.s006.pdf]

Prototypical STARD diagram to report flow of participants through the study

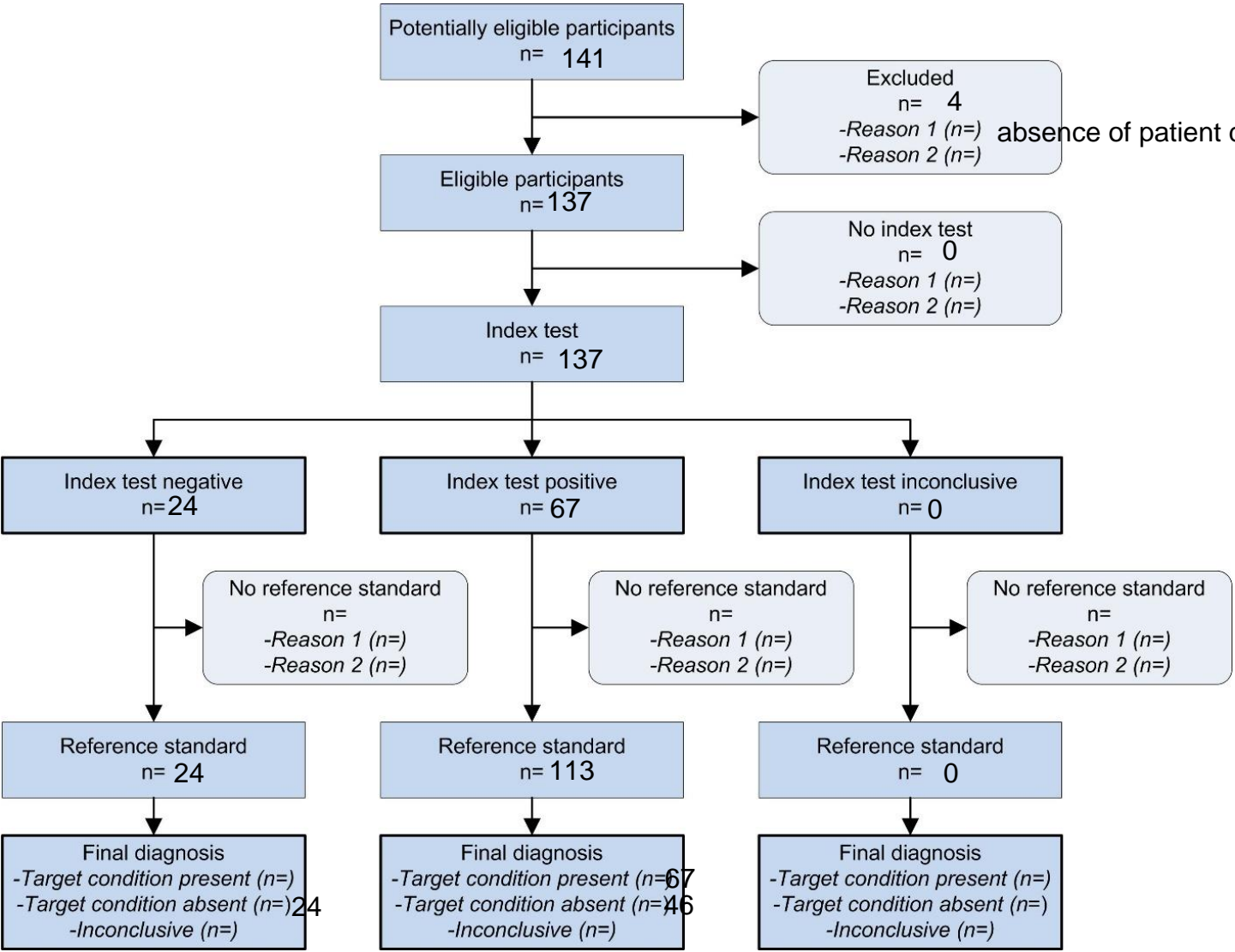

Supplement: S5 STARD — (PDF) [file pntd.0006706.s007.pdf]
