# Supplementary material for: Evaluation of Auramine O staining and conventional PCR for leprosy diagnosis: A comparative cross-sectional study from Ethiopia
Source: PLoS Negl Trop Dis. 2018 Sep 4;12(9):e0006706. doi: 10.1371/journal.pntd.0006706 (PMC6138420; doi:10.1371/journal.pntd.0006706)
Supplement: S3 Table — Statistical value obtained with the binomial tests and the Fisher test for the different tests in different condition (all samples, LB = low bacillary (BT, TT and INT) or HB = high bacillary (LL, BL and BB))–p = p-value in red when p> 0.05 and in green when p<0.05; OR: odds ratio. (DOCX) [file pntd.0006706.s010.docx]

S3 Table: Statistical value obtained with the binomial tests and the Fisher test for the different tests in different condition (all samples, LB=low bacillary (BT, TT and INT) or HB=high bacillary(LL, BL and BB)) – p= p-value in red when p> 0.05 and in green when p<0.05; OR: odds ratio;

|  |  | **All samples** | | | | | **LB samples** | | | | | |
| --- | --- | --- | --- | --- | --- | --- | --- | --- | --- | --- | --- | --- |
|  |  | **ZN** | **AO in SSS** | **FF** | **AO in tissue** | **PCR_m+c_** | **ZN** | **AO in SSS** | **FF** | **AO in tissue** | **PCR** | **PCR_m+c_** |
| **All samples** | **AO in SSS** | ***p*=0.09** |  |  |  |  |  |  |  |  |  |  |
|  | **FF** | ***p*=0.006 - OR= 2.3** | ***p*=0.08 - OR= 1.75** |  |  |  |  |  |  |  |  |  |
|  | **AO in tissue** | ***p*=0.003 - OR= 2.4** | ***p*=0.05 - OR= 1.85** | ***p*=1** |  |  |  |  |  |  |  |  |
|  | **PCR** |  |  | ***p*=0.005 - OR=3** | ***p*=0.009 – OR=2.9** |  |  |  |  |  |  |  |
|  | **PCR_c_** |  |  |  |  | ***p*=0.04 - OR= 5.6** |  |  |  |  |  |  |
| **HB** | **ZN** |  |  |  |  |  | ***p*=1.7e-8 - OR=10.9** |  |  |  |  |  |
|  | **AO in SSS** |  |  |  |  |  |  | ***p*=3.5e-7 - OR=9.9** |  |  |  |  |
|  | **FF** |  |  |  |  |  |  |  | ***p*=3.3e-5 - OR=8.8** |  |  |  |
|  | **AO in tissue** |  |  |  |  |  |  |  |  | ***p*=7.2e-5 - OR=8.2** |  |  |
|  | **PCR** |  |  |  |  |  |  |  |  |  | ***p*=0.007- OR=10.9** |  |
| **LB** | **AO in SSS** |  |  |  |  |  | ***p*=0.2** |  |  |  |  |  |
|  | **FF** |  |  |  |  |  | ***p*=0.0004** | ***p*=0.01** |  |  |  |  |
|  | **AO in tissue** |  |  |  |  |  | ***p*=0.01** | ***p*=0.01** | ***p*=1** |  |  |  |
|  | **PCR** |  |  |  |  |  | ***p*=2.2e-16** | ***p*=9.8e-9** | ***p*=0.01** | ***p*=0.01** |  |  |
|  | **PCR_m+c_** |  |  |  |  |  |  |  |  |  |  |  |
|  | **PCR_c_** |  |  |  |  |  |  |  |  |  |  | ***p*=0.15, OR= 3.7** |
