# Supplementary material for: Evaluation of Auramine O staining and conventional PCR for leprosy diagnosis: A comparative cross-sectional study from Ethiopia
Source: PLoS Negl Trop Dis. 2018 Sep 4;12(9):e0006706. doi: 10.1371/journal.pntd.0006706 (PMC6138420; doi:10.1371/journal.pntd.0006706)

S1 Fig: Raw data used for the calculation of the sensitivity, specificity, PPV and NPV of the routine methods, AO and PCR- Each value represented the number of patients per group


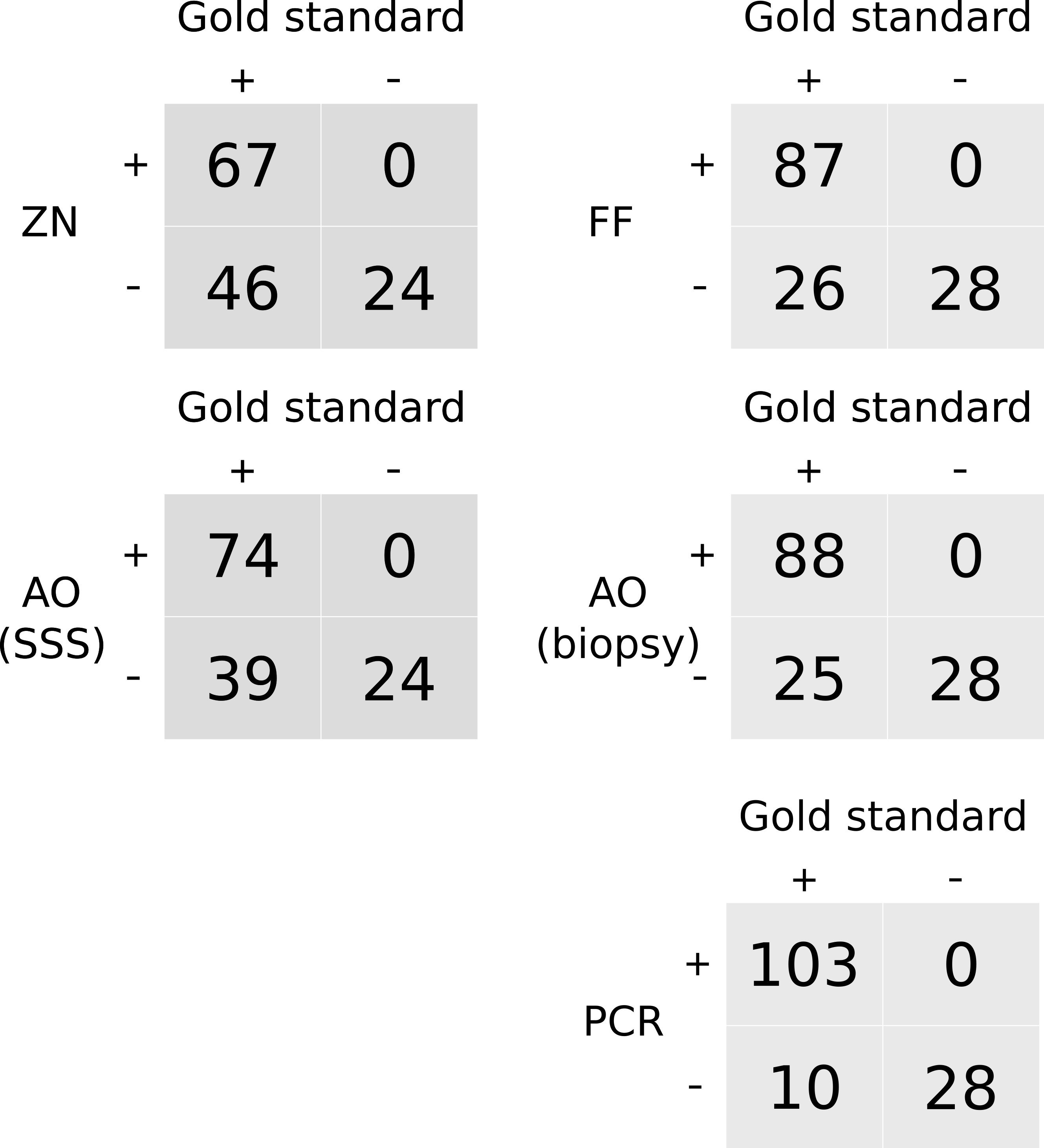

Supplement: S1 Fig — (DOCX) [file pntd.0006706.s011.docx]
