# Supplementary material for: Evaluation of Auramine O staining and conventional PCR for leprosy diagnosis: A comparative cross-sectional study from Ethiopia
Source: PLoS Negl Trop Dis. 2018 Sep 4;12(9):e0006706. doi: 10.1371/journal.pntd.0006706 (PMC6138420; doi:10.1371/journal.pntd.0006706)

S2 Fig: Flowchart of the study design representing the collection of data and samples in addition to the number of sample collected and the methods applied to these samples – HD: host depletion, QIAmp UCP: QIAmp UCP Pathogen Mini kit, QIAmp fast: QIAmp Fast DNA Tissue kit, m+e (red): mechanical and enzymatic digestion, e (blue): enzymatique digestion only


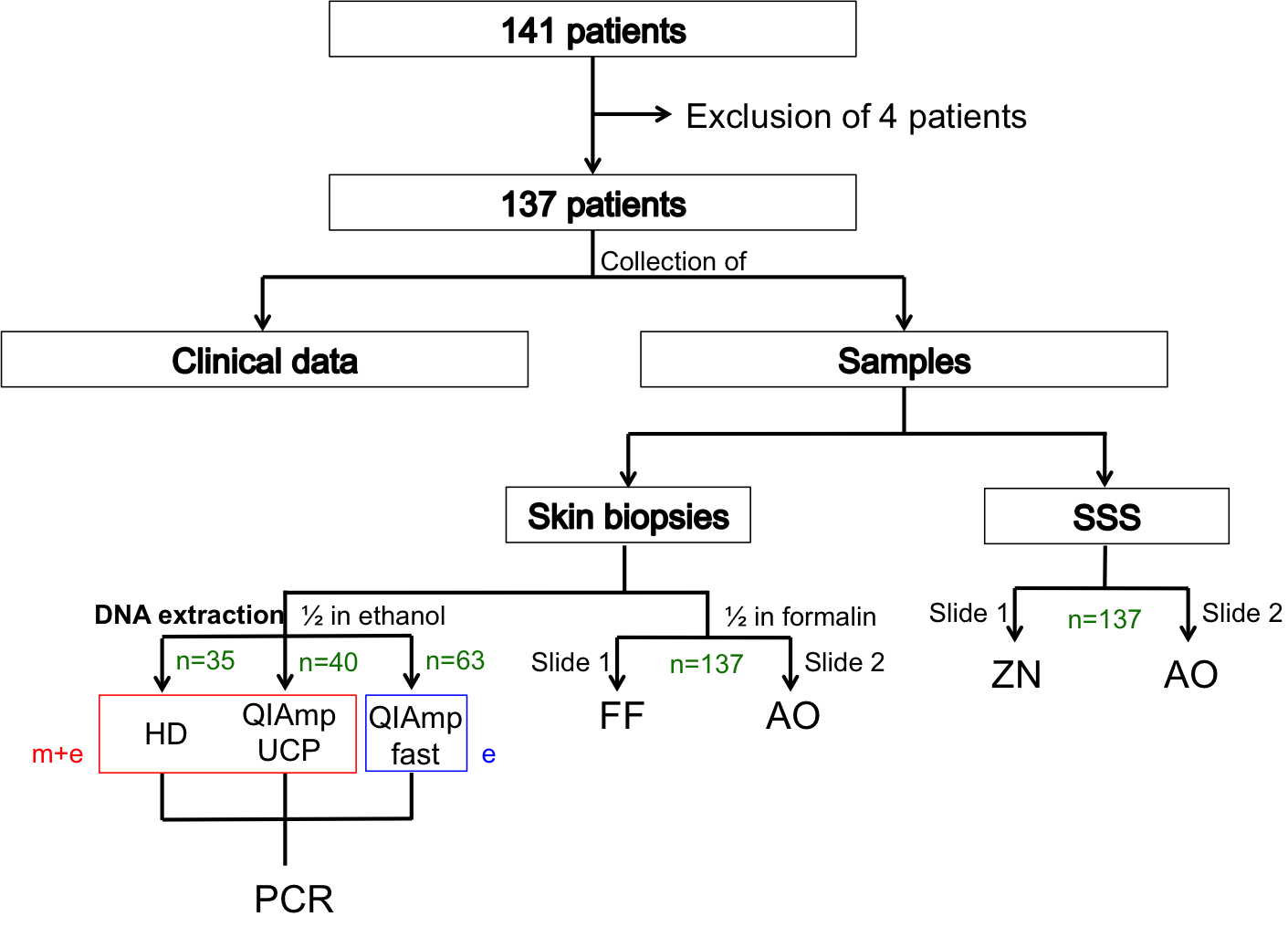

Supplement: S2 Fig — (DOCX) [file pntd.0006706.s012.docx]
